# Screening of core genes and prediction of ceRNA regulation mechanism of circRNAs in nasopharyngeal carcinoma by bioinformatics analysis

**DOI:** 10.3389/pore.2023.1610960

**Published:** 2023-03-28

**Authors:** HongMin Chen, XiaoXiao Shi, Li Ren, YuMing Wan, HongYu Zhuo, Li Zeng, WangMu SangDan, Feng Wang

**Affiliations:** ^1^ Department of Medical Oncology, Cancer Center, West China Hospital, West China, Medical School, Sichuan University, Sichuan, China; ^2^ Department of Medical Oncology, Chengdu Shangjin Nanfu Hospital, West China Hospital, Sichuan University, Chengdu, China; ^3^ Department of Thoracic Oncology, Cancer Center, West China Hospital, Sichuan University, Chengdu, China; ^4^ Department of Oncology, People’s Hospital of Tibet Autonomous Region, Lhasa, China

**Keywords:** TCGA, nasopharyngeal carcinoma, weighted gene co-expression network analysis, GEO database, fibronectin 1

## Abstract

**Background:** Nasopharyngeal carcinoma (NPC) represents a highly aggressive malignant tumor. Competing endogenous RNAs (ceRNA) regulation is a common regulatory mechanism in tumors. The ceRNA network links the functions between mRNAs and ncRNAs, thus playing an important regulatory role in diseases. This study screened the potential key genes in NPC and predicted regulatory mechanisms using bioinformatics analysis.

**Methods:** The merged microarray data of three NPC-related mRNA expression microarrays from the Gene Expression Omnibus (GEO) database and the expression data of tumor samples or normal samples from the nasopharynx and tonsil in The Cancer Genome Atlas (TCGA) database were both subjected to differential analysis and Weighted Gene Co-expression Network Analysis (WGCNA). The results from two different databases were intersected with WGCNA results to obtain potential regulatory genes in NPC, followed by Gene Ontology (GO) and Kyoto Encyclopedia of Genes and Genomes (KEGG) functional enrichment analyses. The hub-gene in candidate genes was discerned through Protein-Protein Interaction (PPI) analysis and its upstream regulatory mechanism was predicted by miRwalk and circbank databases.

**Results:** Totally 68 upregulated genes and 96 downregulated genes in NPC were screened through GEO and TCGA. According to WGCNA, the NPC-related modules were screened from GEO and TCGA analysis results, and the genes in the modules were obtained. After the results of differential analysis and WGCNA were intersected, 74 differentially expressed candidate genes associated with NPC were discerned. Finally, fibronectin 1 (FN1) was identified as a hub-gene in NPC. Prediction of upstream regulatory mechanisms of FN1 suggested that FN1 may be regulated by ceRNA mechanisms involving multiple circRNAs, thereby influencing NPC progression through ceRNA regulation.

**Conclusion:** FN1 is identified as a key regulator in NPC development and is likely to be regulated by numerous circRNA-mediated ceRNA mechanisms.

## Introduction

Nasopharyngeal carcinoma (NPC) is a dominantly prevailing malignant tumor originating from the nasopharyngeal mucosal lining, and the tumor primarily occurs at the pharyngeal recess (1). In 2020, NPC contributes to approximately 133,000 new cases and 80,000 deaths worldwide (2). The 10-year survival rate can reach 98% for stage I NPC patients and 60% for stage II patients, but the median survival is merely 3 years for advanced stages (3). Due to the deep tumor location and absence of obvious clinical manifestations at the early stage, most patients have already advanced to the middle and late stages when diagnosed (4). Despite major advances in modern medicine, NPC remains one of the most difficult tumor types to distinguish and treat (5). At present, the potential modes and mechanisms of NPC development are still unclear. This underscores the need for identifying the molecular mechanisms underlying NPC initiation and development to efficiently diagnose and treat patients.

The functional screening of core genes contributing to cancer progression and structural characterization of cancer genomes provide novel and complementary insights into the essential molecular mechanisms and pathways behind diverse cancer types, which is of great value for the development of personalized therapies (6). Growing evidence unveils the involvement of numerous abnormally expressed non-coding RNAs in the initiation and progression of NPC (7, 8). Dysregulated circRNAs can act as tumor suppressors or oncogenes, controlling cell proliferation, migration, apoptosis, and tumor metastasis (9, 10), highlighting the imperative function of circRNAs in mediating carcinogenesis. Intrinsically, circRNA can exert vital roles as a competitive endovascular RNA (ceRNA) or protein-coding RNA, or interact with RNA-binding proteins to regulate tumor development and gene expression (11, 12). Nevertheless, the research on circRNAs related to NPC development is still scarce, and their potential mechanisms need to be clarified further.

Recently, with the development of molecular biotechnology, bioinformatics analysis has played a key role in screening tumor candidate biomarkers, providing a new direction for the study and prediction of tumor pathogenesis including NPC (13). For instance, Zhu et al. revealed in their work that curcumin can enhance NPC cell sensitivity to radiotherapy by mediating the ceRNA mechanism based on bioinformatics analysis (14). Zhang et al. identified several microRNAs (miRNAs) with potential as prognostic markers for NPC through miRNA microarrays and bioinformatics screening (15). Although researchers have conducted numerous studies on NPC, the core genes related to NPC and their regulatory mechanisms are still poorly understood. Therefore, we aimed to identify the hub-gene in NPC by performing the integrative analysis of Gene Expression Omnibus (GEO) and The Cancer Genome Atlas (TCGA) datasets as well as differential analysis and functional enrichment analysis of NPC-associated genes, so as to provide a new direction for comprehensively understanding the pathogenesis and potential regulatory mechanisms of NPC.

## Materials and methods

### GEO data acquisition and difference analysis

The NPC-related mRNA expression microarrays GSE13597, GSE34573, and GSE53819 (16–18) were downloaded from the GEO database. Among them, GSE13597 included 3 normal samples and 25 tumor samples, GSE34573 included 4 normal samples and 16 tumor samples, and GSE53819 included 18 normal samples and 18 tumor samples. All data were downloaded from the microarray expression data in GEO database. During gene annotation, if a gene corresponded to multiple probes, the average expression value of these probes was selected as the expression value of the gene. The R language “limma” package and “sva” package (19–21) were adopted to merge and batch-correct the data from the three chips (19), and an empirical Bayes framework was used to adjust for batch effects. Subsequently, with normal samples as the controls, differential analysis was performed using the “limma” package, and *p*-value was corrected using the false discovery rate (FDR) method, with |logFC| > 1 and adj. p.value <0.05 as the criteria to screen differentially expressed genes (DEGs). Meanwhile, the NPC-related miRNA expression microarray GSE70970 was obtained from GEO, which included 17 normal samples and 246 normal samples, and then the microarray was differentially analyzed using the “limma” package.

### TCGA data acquisition and difference analysis

The expression data of NPC-related genes were downloaded from the TCGA GDC database. Specifically, only tumor sample expression data and normal sample expression data of selected nasopharyngeal tissues (including the pharynx and tonsil) were downloaded. Subsequently, differential analysis was performed using the R language “limma” package and “edgeR” package (22–24), and normal samples were used as controls. The FDR method was utilized to correct the differential *p*-value, with |logFC| > 1 and adj.p.value <0.05 as screening criteria.

### Weighted gene co-expression network analysis (WGCNA) analysis

The combined GEO gene expression data and TCGA data were analyzed by WGCNA using the R language “WGCNA” package (25), respectively. In brief, genes were divided into modules of different colors by analyzing the association between genes. The genes showing no association with other genes were classified into the gray module (a meaningless module). The module was recognized by dynamic clipping, the minimum number of genes in the module was set to 50, and the recommended soft threshold was automatically calculated by the soft threshold selection program. The similarity between modules was calculated based on eigengenes of modules using the cor function in R language, and the genes with high similarity were merged. Thereafter, the correlation between merged modules and sample phenotype was analyzed.

### Functional enrichment analysis of candidate genes

Gene Ontology (GO) and Kyoto Encyclopedia of Genes and Genomes (KEGG) functional enrichment analyses of identified candidate genes were performed using “clusterprofile,” “org.Hs.eg.db,” “enrichplot,” and “ggplot2” packages. Meanwhile, the functional enrichment bubble diagram was drawn. The *p*-value <0.05 indicated significant enrichment.

### Protein-protein interaction (PPI) analysis

The interaction analysis of candidate genes was performed through the STRING database (https://cn.string-db.org/), and the gene interaction network was constructed using cytoscapev3.9.1. The degree value of each gene was calculated to obtain the hub-gene in the gene interaction network.

### Prediction of upstream regulatory mechanisms of candidate genes

The upstream miRNAs of candidate genes were predicted using miRwalk database (http://mirwalk.umm.uni-heidelberg.de/), and the miRNA-mRNA regulatory network was mapped using cytoscape v3.8.1. The circRNA-miRNA regulation data were downloaded from the circBank database (http://www.circbank.cn/index.html), and the data with a prediction score greater than 500 points were retained for the subsequent prediction of the upstream circRNAs of candidate miRNAs. Subsequently, the circRNA-miRNA-mRNA regulation network diagram was drawn using “ggplot2,” “ggalluvial,” and “RColorBrewer” packages.

## Results

### Screening of DEGs in NPC

**FIGURE 1:**
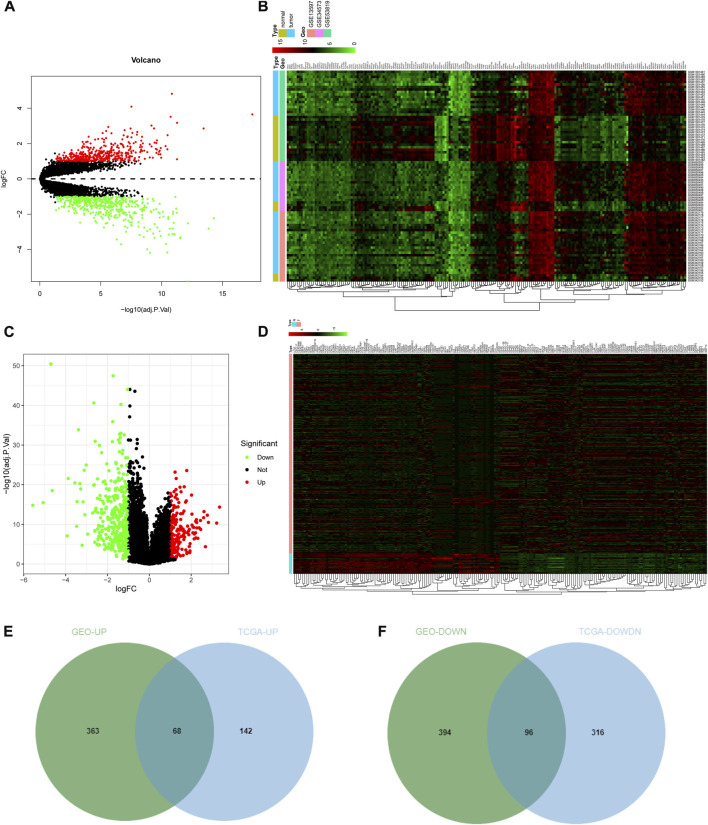
Screening of DEGs. **(A)** Volcano plot of DEGs in GEO database. The abscissa indicated the logFC, the ordinate indicated the −log10 (adj.p.val), red dots in the plot indicated significantly upregulated genes, and green dots indicated significantly downregulated genes in tumor samples; **(B)** Heat map of the top 100 significant DEGs in GEO. The abscissa represented the sample number, the ordinate represented the gene name, and the histogram in the upper right represented the color scale. **(C,D)** Volcano plot and heat map of DEGs from TCGA NPC dataset; **(E,F)** The intersection of significantly upregulated genes **(E)** or the intersection of significantly downregulated genes **(F)** from GEO and TCGA NPC datasets, respectively. The middle part indicated the intersection of the two datasets.

NPC-related expression microarrays GSE13597, GSE34573, and GSE53819 from the GEO database were merged, and the combined data were corrected in batch. Through differential analysis of integrated NPC gene expression data, 921 significant DEGs were identified, of which 431 genes were upregulated and 490 genes were downregulated in NPC samples (Figures 1A, B; Supplementary Table S1). Subsequently, the gene expression data of tumor samples and normal samples from selected tissues (such as the nasopharynx) were downloaded from TCGA database and then differentially analyzed. Consequently, 622 significant DEGs in NPC samples were discerned. Among them, 210 genes were highly expressed and 412 genes were weakly expressed in NPC samples (Figures 1C, D; Supplementary Table S2). To further screen genes that may exert an imperative regulatory role in NPC, the DEGs from GEO database and TCGA database were intersected. The upregulated genes in tumors from GEO analysis were intersected with upregulated genes in tumors from TCGA analysis (Figure 1E), and then 68 genes were identified to be highly expressed in both sets of tumor data, which may have the function of promoting tumorigenesis. Similarly, an intersection of significantly under-expressed genes in NPC (Figure 1F) identified 96 weakly expressed genes in both sets of tumor data, which may have the ability to inhibit tumor development.

### Sample clustering and power value selection in WGCNA

**FIGURE 2:**
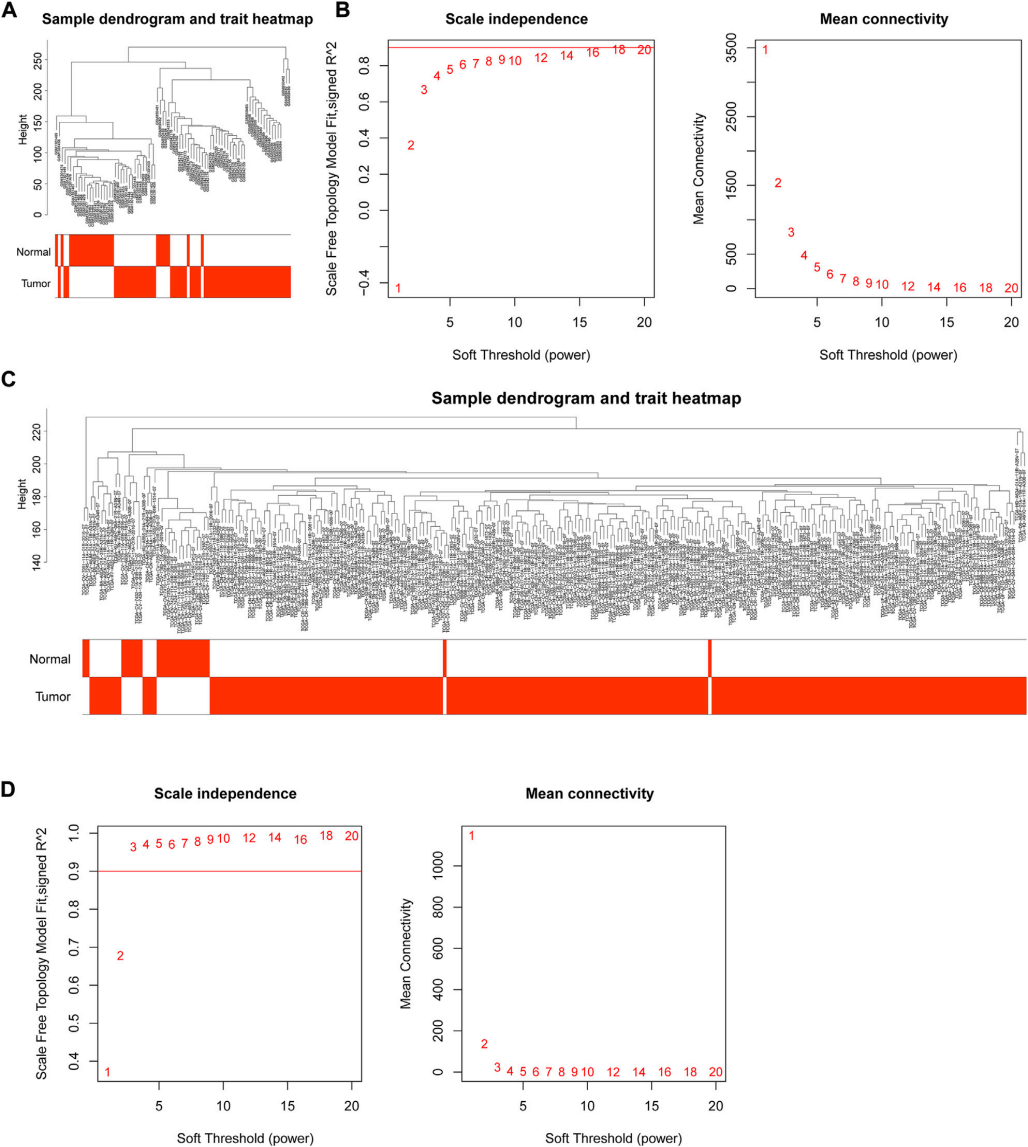
WGCNA of GEO and TCGA data. **(A)** Outlier detection of GEO samples; **(B)** Network topology for different soft threshold powers. The number indicated the corresponding soft threshold power. When soft threshold was 14, approximate scale-free topology could be obtained, indicating that this value was effective for gene partition; **(C)** Outlier detection of TCGA samples; **(D)** Screening of soft thresholds in TCGA data. The approximate scale-free topology could be acquired when soft threshold was 3.

Through WGCNA, genes could be grouped and divided into multiple different modules to understand the correlation between genes and clinical traits. The integrated GEO data and TCGA data were subjected to outlier detection of samples using the “WGCNA” package in R language (Figures 2A, C), and there were no obvious outliers. All samples were retained for subsequent analyses. In an attempt to identify gene modules associated with NPC, GEO and TCGA data module classifications were subjected to screening of soft thresholds, which showed that the optimal soft threshold was 14 in GEO data and the optimal soft threshold was 3 in TCGA data (Figures 2B, D). These two soft thresholds were chosen for subsequent gene module partition.

### Module clustering in WGCNA

**FIGURE 3:**
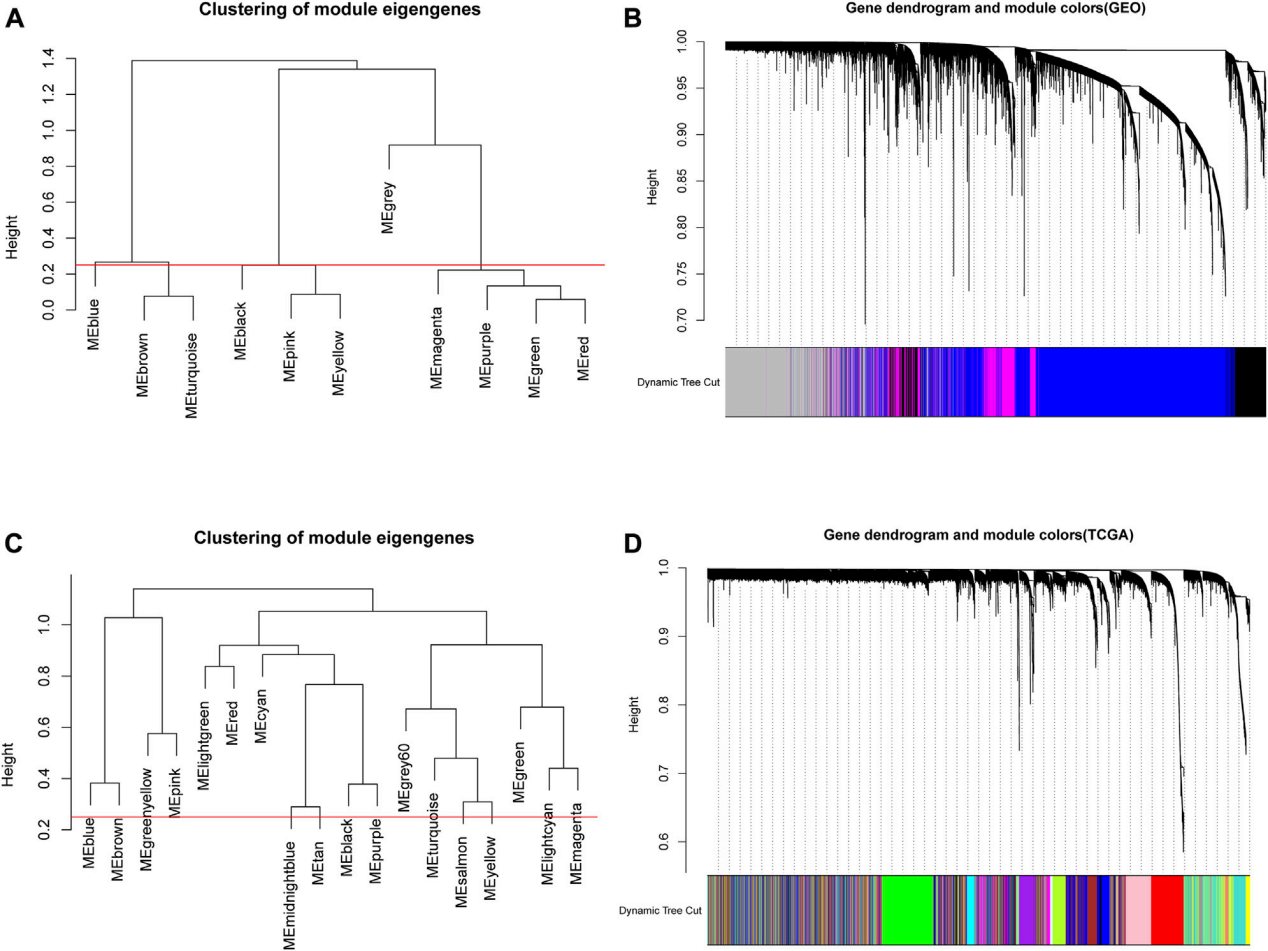
Module clustering in WGCNA. **(A,C)** The correlation analysis of modules prior to the merging of GEO and TCGA data. The ordinate indicated the similarity between modules and the low value indicated high similarity. Red lines indicated the shear height and 0.25 was selected as the shear height in this study (i.e., modules with similarity above 0.75 were merged); **(B,D)** Gene dendrogram of genes in corresponding modules in GEO and TCGA databases.

Based on the above analysis, the appropriate soft thresholds were selected for module partition of genes in GEO and TCGA data, respectively. The modules with similarity greater than 0.5 were merged, where gray modules were non-sense modules and genes in this module were mutually unrelated genes. In GEO data, a total of 11 gene modules of different colors were obtained (Figure 3A), and 4 modules of different colors were retained after merging (Figure 3B). Through the same approach, the genes in TCGA were subjected to module partition. A total of 18 gene modules with different colors were obtained (Figure 3C), and 18 modules with different colors remained after merging (Figure 3D).

### Correlation analysis of WGCNA modules

**FIGURE 4:**
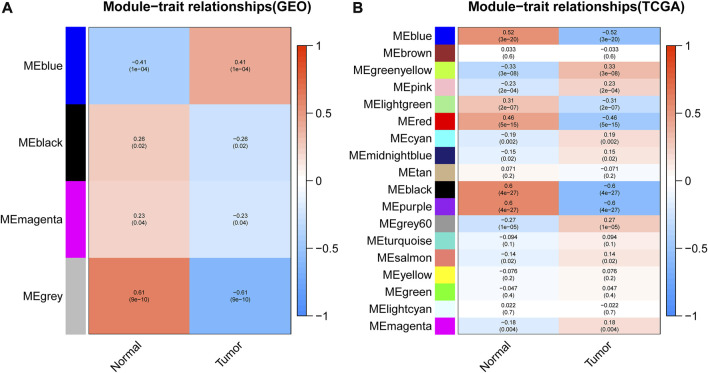
Correlation analysis between modules and clinical traits. **(A,B)** represented the correlation analysis between the modules obtained from the WGCNA analysis of GEO data or TCGA data with sample traits, respectively. The abscissa represented the trait type and different colors represented different correlations and *p* values.

After gene module partition, further clinical correlation analysis was performed on the WGCNA results of GEO data and TCGA data, respectively. In GEO analysis results, the correlation analysis between 4 gene modules and clinical traits revealed that the blue module was prominently positively correlated with tumors (Figure 4A; Supplementary Table S3), which implied that the genes in the blue module may promote tumor development. Based on the TCGA database, the black module and purple module were negatively correlated with the occurrence of tumors (Figure 4B; Supplementary Tables S4, S5), which indicated that the genes in these two modules may confer an inhibitory effect on the occurrence and development of tumors.

### Screening of candidate genes in NPC

**FIGURE 5:**
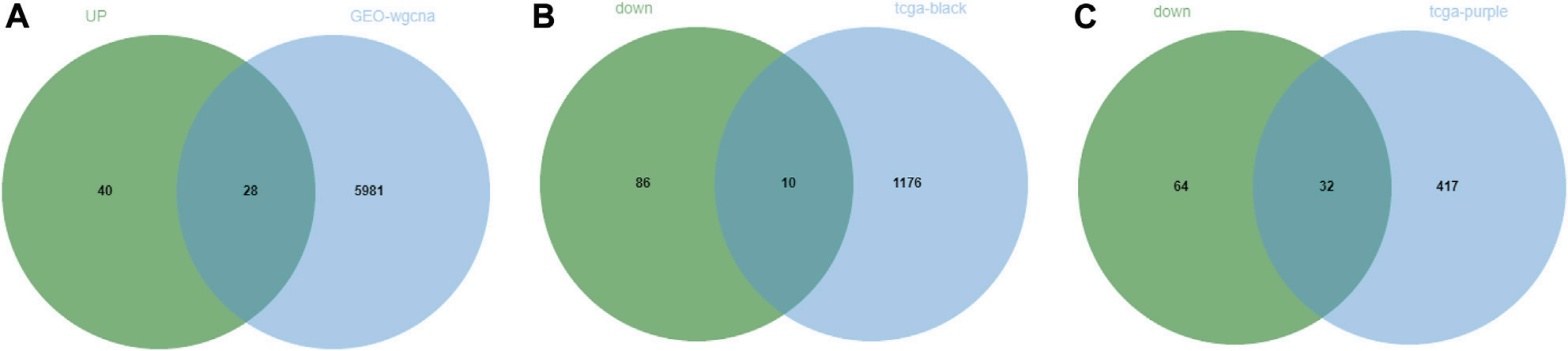
Screening of candidate genes in NPC. **(A)** The intersection between significantly highly expressed genes in NPC obtained from GEO database or TCGA database with genes in the module significantly positively correlated with tumor obtained by WGCNA of GEO data; **(B,C)** The intersection between significantly lowly expressed genes in NPC obtained from GEO database or TCGA database with genes in two modules significantly negatively correlated with tumor obtained by WGCNA of TCGA data.

Through the above differential analysis, 68 genes that were highly expressed in tumors were identified, which may have potential promoting effects on tumor development. Meanwhile, based on WGCNA, we found that the genes in the blue module may also have a tumor-promoting effect. Thereafter, an intersection of these two sets of genes was taken (Figure 5A) and consequently 32 candidate genes were identified, which may be crucial contributing factors to tumor development. Similarly, the 96 genes with significantly low expression in tumors were respectively intersected with genes in the two modules that were significantly negatively correlated with tumors obtained by WGCNA (Figures 5B, C), and finally, 10 and 32 candidate genes were discerned, respectively. These 42 genes may be suppressors of tumor development. In summary, we identified 74 DEGs (32 tumor-promoting genes and 42 tumor-suppressive genes) that were extensively associated with NPC as candidate genes.

### Enrichment analysis

**FIGURE 6:**
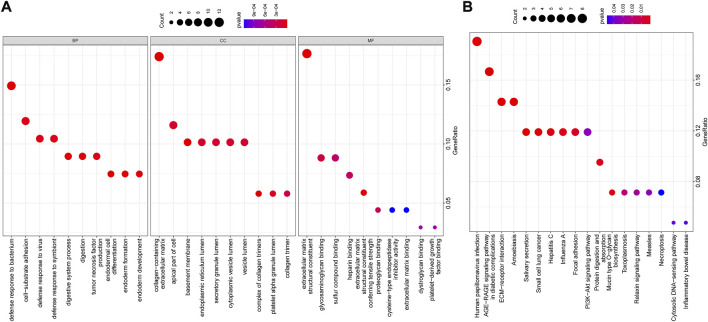
**(A,B)** GO and KEGG functional enrichment analyses of candidate genes. The abscissa indicated the GenerRatio, the ordinate indicated the functional item, circle size indicated the number of enriched genes, color indicated the enrichment p-value, and left histogram indicated the color scale.

GO and KEGG functional enrichment analyses were further performed on the above 74 candidate genes. GO functional enrichment analysis revealed that these genes were enriched in relevant items such as “defense response to bacterium,” “collagen-containing extracellular matrix,” and “extracellular matrix structural constituent” (Figure 6A), indicating the underlying role of these functional items in NPC progression. KEGG pathway enrichment analysis unveiled that these genes were mainly enriched in related signaling pathways such as “human papillomavirus (HPV) infection” and “advanced glycation endproduct (AGE)-receptor for AGE (RAGE) signaling pathway in diabetic complications” (Figure 6B), suggesting that these signaling pathways may be one of the reasons for the development of NPC.

### PPI analysis

**FIGURE 7:**
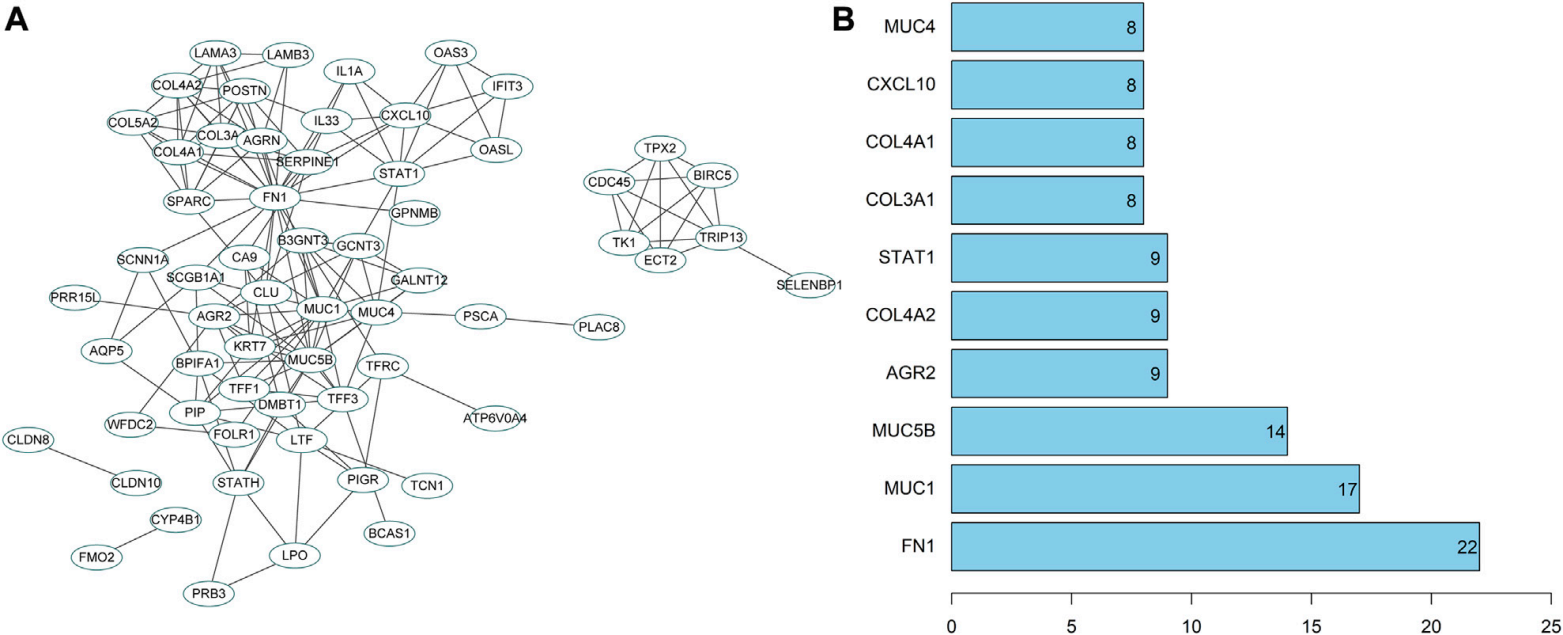
PPI analysis of candidate genes. **(A)** The interaction analysis of candidate genes. Each ellipse represented a gene, and the line between genes indicated the presence of interaction relation between genes; **(B)** The degree value of the top 10 genes. The abscissa represented the degree value, and the ordinate represented the gene name.

To further screen potential regulatory genes in NPC, 74 candidate genes were subjected to interaction analysis, and a gene interaction network was constructed (Figure 7A). The interaction between genes was also analyzed to obtain the degree value of each gene (Figure 7B). The results revealed that the degree values of the genes in the top 10° values were all ≥8, indicating that there may be an interactive relationship between these 10 genes and at least 8 other genes. Importantly, the degree value of fibronectin 1 (FN1) was the largest, indicating that FN1 was the core in the gene interaction network and a hub-gene among these candidate genes. Therefore, FN1 may exert a crucial role in the development of NPC.

### ceRNA prediction of FN1 gene

**FIGURE 8:**
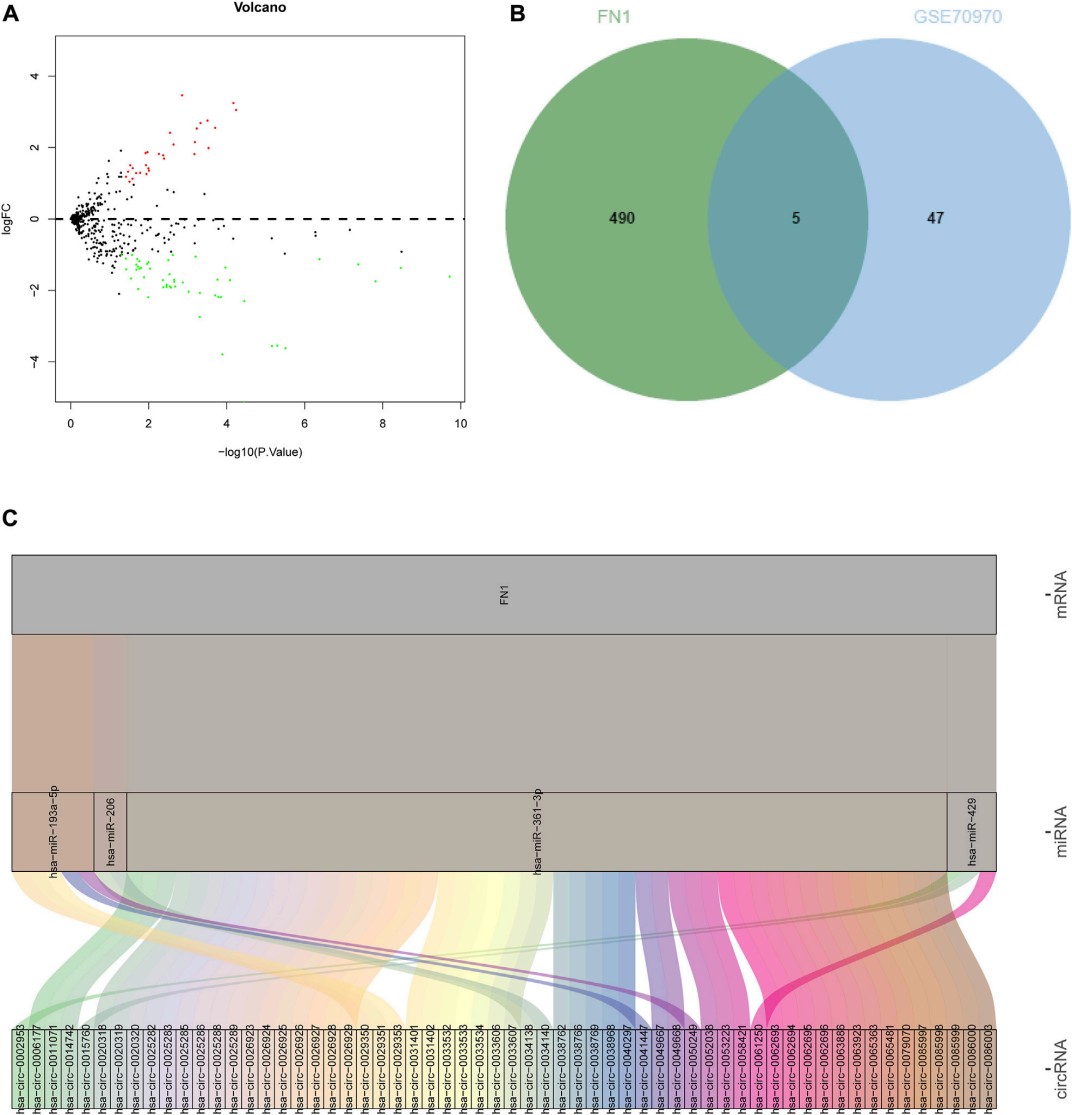
Prediction of upstream ceRNA mechanisms of the hub-gene. **(A)** Volcano plot of NPC-related miRNA microarray; **(B)** The intersection of the predicted upstream miRNAs of the hub-gene and significantly differentially expressed miRNAs in the microarray. The middle part indicated the intersection of the two groups of data; **(C)** Prediction of upstream circRNAs of the hub-gene. The leftmost part represented the circRNA, the middle represented the miRNA, the right represented the mRNA, and the line between the three represented a predicted regulatory relationship between them.

The differential expression of the identified hub-gene FN1 from GEO and TCGA was retrieved (Table 1), which revealed that FN1 was significantly upregulated in tumor samples. Meanwhile, a NPC-related miRNA expression microarray GSE70970 was acquired from GEO database. Differential analysis of miRNA expression in this microarray identified 29 upregulated miRNAs and 52 downregulated miRNAs in NPC (Figure 8A; Supplementary Table S6). Subsequently, the upstream miRNAs of FN1 were predicted, and the results were intersected with the significantly downregulated miRNAs in the microarray (Figure 8B), thus screening 5 potential upstream regulatory miRNAs. Further upstream circRNAs of the 5 miRNAs were predicted, 4 of which were involved in circRNA-mediated ceRNA regulation, and 60 potentially regulatory circRNAs were identified (Figure 8C; Supplementary Table S7), which meant that FN1 was very likely regulated by multiple circRNA-mediated ceRNA mechanisms, thus affecting the progression of NPC.

**TABLE 1:** Differential expression of the hub-gene.

Symbol	GEO-logFC	GEO-adjp	TCGA-logFC	TCGA-adjp
FN1	2.35	2.96E-08	2.14	4.57E-07

## Discussion

NPC is chiefly characterized by recurrence and distant metastasis, making it an extremely life-threatening cancer worldwide (26). To make matters worse, NPC is difficult to identify and treat clinically because of early non-specific symptoms (27–29). Therefore, a better understanding of the mechanisms of NPC occurrence and progression and the identification of reliable markers of NPC are essential for the clinical diagnosis and treatment of NPC. In this study, we integrated three NPC-related microarrays from GEO database and retrieved the NPC samples included in TCGA. Subsequently, differential analysis and WGCNA of the obtained gene expression data were conducted to identify crucial genes associated with NPC, and their potential regulatory mechanisms were also preliminarily predicted.

It is noteworthy that increased oncogene expression and decreased tumor suppressor gene expression are indeed key factors in tumorigenesis (30). With the development of sequencing technology and chip technology, we can easily screen the expression levels of thousands of genes in the human genome simultaneously (30). In particular, WGCNA is an effective method for gene grouping through gene co-expression relationships to understand the association between genes and clinical traits (25), which is utilized to identify core modules and hub-genes associated with diabetic nephropathy (31) and breast cancer (32). In our current study, through differential analysis and WGCNA of NPC-related gene expression from GEO and TCGA datasets, 74 candidate genes were screened.

Furthermore, according to GO analysis, these genes unleashed crucial roles in extracellular matrix structural constituent, collagen-containing extracellular matrix, and defense response to bacterium. The extracellular matrix, composed of intercellular substance and basement membrane, functions as an essential tissue barrier for carcinoma metastasis, and cancer cells can degrade the matrix through secretion or activation of protein-degrading enzymes following their surface receptors’ adhesion to various components of extracellular matrix, thus constituting channels for metastasis (33). Prior evidence has indicated a distinct change in the extracellular matrix remodeling pathway in NPC tissues (34). On the other hand, KEGG pathway enrichment analysis of these candidate genes showed that the main enriched pathways included the HPV infection and AGE-RAGE signaling pathway in diabetic complications. Viral infections, in particular HPV and Epstein-Barr virus (EBV), are considered crucial etiological agents for NPC development, and HPV infection rate in NPC has been reported to range from 9% to 52.9%, largely depending on the geographic distribution and ethnicity of the population and viral detection methods (35). It is interesting to note that patients with HPV-positive NPC are afflicted by larger primary tumors and greater local symptoms than those with EBV-positive NPC (36). The interaction between S100P and RAGE potentiates C666-1 cell proliferation and migration, and S100P-RAGE blockade is a potential therapeutic modality for NPC (37, 38). Therefore, GO and KEGG analyses can greatly help identify cellular components, molecular functions, biological processes, and crucial pathways underlying the occurrence and progression of NPC.

Further PPI analysis of 74 candidate genes revealed that the FN1 gene occupied a core position in the gene interaction network. FN1 is a glycoprotein that upregulates expression levels of matrix metalloproteinases to promote cancer cell local invasion and distant metastasis, and its overexpression in NPC is closely related to an advanced stage and poor survival (39). Of note, upregulation of FN1 impedes apoptosis by activating the NF-κB pathway and is also involved in facilitating proliferation, invasion, migration, and epithelial-mesenchymal transition in NPC cells (40, 41). These existing studies have further confirmed the role of FN1 as a hub gene in NPC.

The interaction between circRNAs and miRNAs has significant influences on key genes, thus disturbing the development of cancer (42, 43). Recently, the roles of various non-coding RNAs in mediating diverse biological pathways and functions in NPC have been extensively documented (44). Compelling evidence has revealed that circRNAs intrinsically mediate tumorigenesis and tumor cell proliferation and migration by competitively binding to miRNAs, thus participating in the pathogenesis of NPC (45, 46). Intriguingly, evidence suggests that FN1 serves as a direct transcriptional target of multiple miRNAs in tumors and circ0081534 has been pointed out to exert tumor oncogenic functions by manipulating the miR-508-5p/FN1 axis in NPC (47). Elevation of miR-9-3p suppresses the malignant behaviors of NPC cells by downregulating FN1 (40). Therefore, the potential upstream regulatory mechanisms of FN1 were predicted subsequently to provide a new research direction and theoretical basis for further understanding the detailed mechanisms of FN1 in NPC.

Although FN1 was previously studied in NPC, we further clarified the role and function of FN1 in the regulation of NPC through a comprehensive analysis of large NPC data from GEO and TCGA in this study. In addition, the detailed regulatory mechanism of FN1 in NPC was poorly studied. In this study, through differential analysis and WGCNA analysis of NPC-related gene expression, FN1 was finally identified as a possible key regulator in NPC. Meanwhile, the ceRNA axis controlled by multiple circRNAs and miRNAs upstream of FN1 was uncovered. This study provides a new research direction and theoretical references for further understanding the detailed mechanism of FN1 in NPC. Nevertheless, the selected microarray dataset is limited, probably leading to low statistical power and the identification of hub genes merely through bioinformatics is insufficient, and where FN1 is expressed in cells and its association with viruses are elusive. Additionally, the main finding of our study lacks clinically relevant information. The expression and function of FN1 in NPC warrant further experimental verification. It is of utmost importance to further delve into the mechanism of FN1 at the molecular, cellular, and biological levels by conducting molecular biological experiments. Additionally, other potential hub genes are worth exploring for the optimal management of NPC.

## Conclusion

The FN1 gene was screened as a hub-gene by downloading NPC data from GEO and TCGA datasets and using WGCNA analysis and functional enrichment analysis. FN1 gene may be a key regulator in NPC development and regulated by ceRNA mechanisms involving multiple different circRNAs.

## Data Availability

The raw data supporting the conclusion of this article will be made available by the authors, without undue reservation.
